# 24-Hour Efficacy and Ocular Surface Health with Preservative-Free Tafluprost Alone and in Conjunction with Preservative-Free Dorzolamide/Timolol Fixed Combination in Open-Angle Glaucoma Patients Insufficiently Controlled with Preserved Latanoprost Monotherapy

**DOI:** 10.1007/s12325-016-0448-9

**Published:** 2016-12-02

**Authors:** Anastasios-Georgios Konstas, Konstadinos G. Boboridis, Paraskevas Kapis, Konstantinos Marinopoulos, Irini C. Voudouragkaki, Dimitrios Panayiotou, Dimitrios G. Mikropoulos, Eirini Pagkalidou, Anna-Bettina Haidich, Andreas Katsanos, Luciano Quaranta

**Affiliations:** 1 1st University Department of Ophthalmology, Aristotle University of Thessaloniki, Thessaloniki, Greece; 2 3rd University Department of Ophthalmology, Aristotle University of Thessaloniki, Thessaloniki, Greece; 3 Department of Hygiene, Aristotle University of Thessaloniki, Thessaloniki, Greece; 4 Department of Ophthalmology, University of Ioannina, Ioannina, Greece; 5 Department of Medical and Surgical Specialties, Section of Ophthalmology, University of Brescia, Brescia, Italy

**Keywords:** BAK, Dorzolamide/timolol fixed combination, Latanoprost, OSD, Preservative-free tafluprost, 24-h efficacy

## Abstract

**Introduction:**

The aim of the present study was to evaluate the 24-h efficacy, tolerability, and ocular surface health with preservative-free (PF) tafluprost and a PF triple drug regimen comprising tafluprost and dorzolamide/timolol fixed combination (DTFC) in open-angle glaucoma patients who were insufficiently controlled with preserved branded or generic latanoprost monotherapy and who exhibited signs or symptoms of ocular surface disease (OSD).

**Methods:**

Prospective, observer-masked, crossover, comparison. Eligible consecutive open-angle glaucoma patients were randomized to either PF tafluprost or the triple PF regimen for 3 months. They were then crossed over to the opposite therapy for another 3 months. At the end of the latanoprost run-in period and after each PF treatment period, patients underwent habitual 24-h intraocular pressure (IOP) monitoring with Goldmann tonometry in the sitting position (at 10:00, 14:00, 18:00, and 22:00) and Perkins tonometry in the supine position (at 02:00 and 06:00). Tolerability and selected ocular surface parameters were evaluated at baseline and the end of each treatment period.

**Results:**

Forty-three open-angle glaucoma patients completed the trial. Mean 24-h IOP on preserved latanoprost was 22.2 ± 3.9 mmHg. Compared with latanoprost monotherapy, PF tafluprost obtained a greater reduction in mean, peak, and fluctuation of 24-h IOP including the 02:00 and 06:00 time points (*P* < 0.05). With the exception of 24-h fluctuation, the triple PF regimen provided significantly lower IOP parameters than latanoprost or PF tafluprost (*P* < 0.001). Finally, PF tafluprost therapy displayed significantly improved tear film break-up times (6.7 vs 6.0 s), corneal staining (1.3 vs 2.2), and Schirmer I test results (9.1 vs 8.2 mm) compared with the preserved latanoprost baseline (all *P* < 0.01). The triple PF regimen demonstrated similar tear film break-up times (6.1 vs 6.0 s) and Schirmer I test results (8.2 vs 8.2 mm) to latanoprost, but revealed a significant improvement in the corneal stain test (1.7 vs 2.2; *P* < 0.001).

**Conclusions:**

In this trial PF tafluprost therapy provided statistically greater 24-h efficacy and improved tolerability compared with preserved latanoprost. The combination of PF tafluprost and PF dorzolamide/timolol fixed combination was statistically and clinically more efficacious than both monotherapies and demonstrated similar ocular surface characteristics to preserved latanoprost monotherapy.

**Trial registration:**

ClinicalTrials.gov (NCT02802137).

**Funding:**

Santen.

## Introduction

A meaningful intraocular pressure (IOP) reduction remains the mainstay of current glaucoma management [1–3]. Although therapy ideally commences with a monotherapy agent, inevitably monotherapies will not suffice for most patients in the long term [3]. Therefore, stepwise medical therapy is often necessary [1, 3]. It has been established, however, that combined antiglaucoma therapy can adversely influence adherence, tolerability, and ocular tissue health [4–6]. These parameters markedly reduce the success of long-term medical therapy [6, 7]. Treatment advances have been introduced to facilitate the success of combined therapy in real life. For instance, fixed combinations (FCs) were introduced to enhance convenience by minimizing the number of daily drops, improve adherence, enhance tolerability, and conceivably improve long-term ocular outcomes [1, 8–10].

In glaucoma a direct consequence of lifelong combined medical therapy is the cumulative toxic effect of preservatives upon and within ocular tissues [10]. The most common preservative contained in glaucoma medications is benzalkonium chloride (BAK), a quaternary ammonium salt that acts as a detergent by disrupting lipid membranes and denaturing proteins [11]. There is convincing scientific evidence to suggest that BAK elicits substantial toxic damage upon the ocular surface [11–14]. A range of BAK-related ocular surface findings include tear film instability [14, 15], corneal and conjunctival epithelial apoptosis [15], increased tear osmolarity [16], and meibomian gland dysfunction [10]. These signs of ocular surface disease (OSD) cause a variety of ocular symptoms that adversely impact quality of life and ultimately reduce long-term adherence and success of glaucoma therapy [17, 18]. Moreover, chronic exposure to preservatives (especially BAK) may elicit ocular tissue inflammatory and fibrotic reactions that can undermine the potential long-term success of future glaucoma surgery [19–21]. Lastly, there is growing suspicion that BAK can also damage deeper ocular tissues (e.g., the trabecular meshwork) [10, 22]. Importantly, preservative-related ocular tissue toxicity is cumulative; therefore, patients receiving combined therapies with multiple preserved drops over a long period may be particularly prone to OSD [10]. It is now well documented that the majority of chronically treated glaucoma patients exhibit signs or symptoms of OSD [12–14]. Simplifying stepwise therapy by employing FCs and switching when possible to preservative-free (PF) medications may ameliorate these toxic effects and enhance the success of long-term stepwise therapy.

Branded or generic latanoprost 0.005% containing BAK is currently the most popular first-choice monotherapy in Europe for patients with ocular hypertension or glaucoma [23]. However since the branded latanoprost formulation contains a high concentration of BAK (0.02%) [24], there will be, over time, a growing number of patients with signs/symptoms of OSD. The problem may become more acute when latanoprost-treated patients with signs or symptoms of OSD require adjunctive therapy. Currently there is a paucity of clinical information on the impact upon 24-h IOP efficacy and the comparative ocular surface damage, assessed with validated ocular surface metrics, when we switch patients with signs or symptoms of OSD from preserved latanoprost to a PF medication. Established PF treatment options include tafluprost 0.0015% and dorzolamide 2%/timolol 0.5% fixed combination (DTFC). To the best of our knowledge there is no published evidence on the 24-h efficacy, tolerability, and ocular surface health with the use of a triple PF therapy in glaucoma. Therefore, the main objective of the current study was to evaluate the 24-h efficacy and ocular surface health of PF tafluprost and a triple PF regimen (tafluprost and DTFC) in open-angle glaucoma patients insufficiently controlled on branded or generic latanoprost preserved with BAK who also exhibit signs or symptoms of OSD.

## Methods

### Patients

All procedures followed were in accordance with the ethical standards of the responsible committee on human experimentation (institutional and national) and with the Declaration of Helsinki (1964), as revised in 2013. The study was also approved by the University Bioethics Committee. Written informed consent was obtained from all participants prior to any study-related procedure. The study was registered with ClinicalTrials.gov (NCT02802137).

This was a 3-month prospective, observer-masked, crossover, comparative study conducted at an academic glaucoma service. The trial enrolled consecutive patients with signs or symptoms of OSD and early-to-moderate open-angle glaucoma (primary open-angle, exfoliative, or pigmentary glaucoma) who were insufficiently controlled after at least 3 months of therapy with branded or generic BAK-preserved latanoprost monotherapy and demonstrated a latanoprost-treated morning (10:00 ± 1 h) IOP greater than 20 mmHg and at least 20% IOP reduction from untreated baseline in two separate visits. To be considered for inclusion, glaucoma patients must have shown an untreated morning IOP between 25 and 39 mmHg at 10:00 (±1 h) in the clinic. Additional eligibility criteria were age between 21 and 85 years; mild to moderate glaucomatous disc damage and visual field loss (less than −12 dB mean deviation visual field loss attributed to glaucoma and 0.8 or better/less vertical cup-to-disc ratio), and visual acuity better than 0.1 in the study eye. The diagnosis of open-angle glaucoma was made by a senior glaucoma expert (AGK) on the basis of the European Glaucoma Society criteria [1]. Study patients had to exhibit reliable perimetry (at least two visual fields with less than 20% fixation losses, false positives, or false negatives in both eyes) [25] and be able to understand study instructions, comply with study medication usage, and be willing to attend all follow-up visits. A comprehensive clinical examination that included slit-lamp biomicroscopy, Goldmann applanation tonometry, gonioscopy, dilated fundoscopy with a 60-diopter lens, ultrasound pachymetry, and Humphrey 24-2 SITA Standard visual field testing (Carl Zeiss Meditec, Dublin, CA) was performed prior to enrollment.

Exclusion criteria were a previous history of less than 10% IOP decrease on any antiglaucoma medication; signs of ocular infection (except blepharitis); history of inadequate adherence; intolerance or contraindication to either prostaglandins, β-blockers, dorzolamide, or BAK; severe OSD, intraocular conventional or laser surgery in the study eye (within 6 months prior to enrollment); previous history of ocular trauma; use of oral or topical corticosteroids (within 3 months before the enrollment), and use of contact lenses. Additional exclusion criteria were clinical evidence of inflammation, signs of any corneal abnormality precluding reliable IOP measurements, and unwillingness to participate in the trial. Women of childbearing potential and lactating mothers were also excluded.

Eligible participants first underwent a latanoprost-treated 24-h IOP assessment together with an ocular surface evaluation (as described in a following section). Study patients were then randomized to either PF tafluprost monotherapy (Saflutan^®^, Santen Oy, Tampere, Finland) dosed in the evening (21:00) or PF tafluprost administered once in the evening (21:00) and preservative-free DTFC (Cosopt PF^®^, Santen, Santen Oy, Tampere, Finland) dosed twice daily (08:00 and 20:00). A 1-h deviation from prescribed administration time was allowed for the instillation of study medications. After 3 months (±2 weeks) of therapy all participants underwent a second, treated 24-h IOP assessment and a second ocular surface evaluation. Study participants were then switched to the opposite regimen and after another 3 months (±2 weeks) of therapy they underwent a final treated evaluation of 24-h IOP and ocular surface status. Instructions regarding correct eye-drop instillation and adherence were repeated at every visit.

### Procedures

All study patients underwent habitual 24-h IOP monitoring (i.e., with Goldmann applanation tonometry measuring sitting IOP at 10:00, 14:00, 18:00, 22:00 and with Perkins tonometry measuring supine IOP values at 02:00 and 06:00). Nighttime supine IOP measurements were performed 5 min after the patients were awakened. The same masked investigators performed all IOP measurements using the same calibrated instruments. A comprehensive clinical examination was performed at all visits. Ocular surface health was evaluated before any IOP measurement using well-established clinical signs (described below) in accordance with the guidelines and methodology proposed by the International Dry Eye Workshop and Meibomian Gland Dysfunction Workshop [26, 27]. Additionally, patient-reported complaints and symptoms as well as investigator-noted adverse events were recorded at the end of each treatment period.

### Ocular Surface Assessment

After recording any self-reported ocular surface symptoms, we performed the following tests:

1. Tear film break-up time (TFBUT). A small quantity of fluorescein was instilled into the inferior fornix with the use of a fluorescein-impregnated paper strip soaked with a drop of unpreserved normal saline. After a few blinks the patient was instructed to keep the eyelids open and the interval between the last complete blink and the first appearance of a dry spot, or disruption of the tear film viewed with the use of a cobalt blue filter, was recorded.
2. Corneal fluorescein staining. Following the TFBUT test, the cornea was examined for punctate epitheliopathy staining with fluorescein. The pattern and density of the spots were evaluated with the van Bijsterveld grading method using a range of 0–3 [28].
3. Schirmer I test (without anesthesia). The test provides an estimation of reflex tear flow stimulated by the insertion of a filter paper into the conjunctival sac at the junction between the lateral and middle third of the lower eyelid. The length of paper in millimeters soaked by tears within 5 min was recorded in each case.

### Statistics

The primary efficacy endpoint of this trial was the mean 24-h IOP (the average pressure for the six time points). The individual time points, peak, trough, and fluctuation of 24-h IOP were considered secondary endpoints. A mixed model was used for the crossover repeated measures design to adjust for period and carry-over effects. Period and sequence were included in the model as fixed effects. Patients within a sequence were included in the model as a random effect. A 95% confidence interval (CI) was constructed for the adjusted difference in means. An intention-to-treat approach was adopted and the subjects were analyzed according to their randomized group.

The current 24-h study had an 80% power to identify a 1.25-mmHg difference between individual time points and between mean 24-h pressure readings assuming a standard deviation of 2.8 mmHg between treatments if 42 patients completed the trial. When both eyes qualified for inclusion, the worse eye (i.e., the one with the higher IOP at baseline) was selected. Mean 24-h IOP fluctuation (average of the highest minus the lowest IOP for each individual patient) as well as the mean peak and trough pressures were analyzed by the paired *t* test. Ocular surface signs after each treatment period were compared with the paired *t* test. Adverse events were evaluated using Cochran’s* Q* and McNemar’s tests. The Bonferroni-adjusted *P* values are reported to correct for multiple comparisons in secondary endpoints. All other reported *P* values are two-tailed with *P* < 0.05 considered significant. Analyses were conducted using the IBM/SPSS Statistics Release 20.0 software package.

## Results

### Patients

**Fig. 1:**
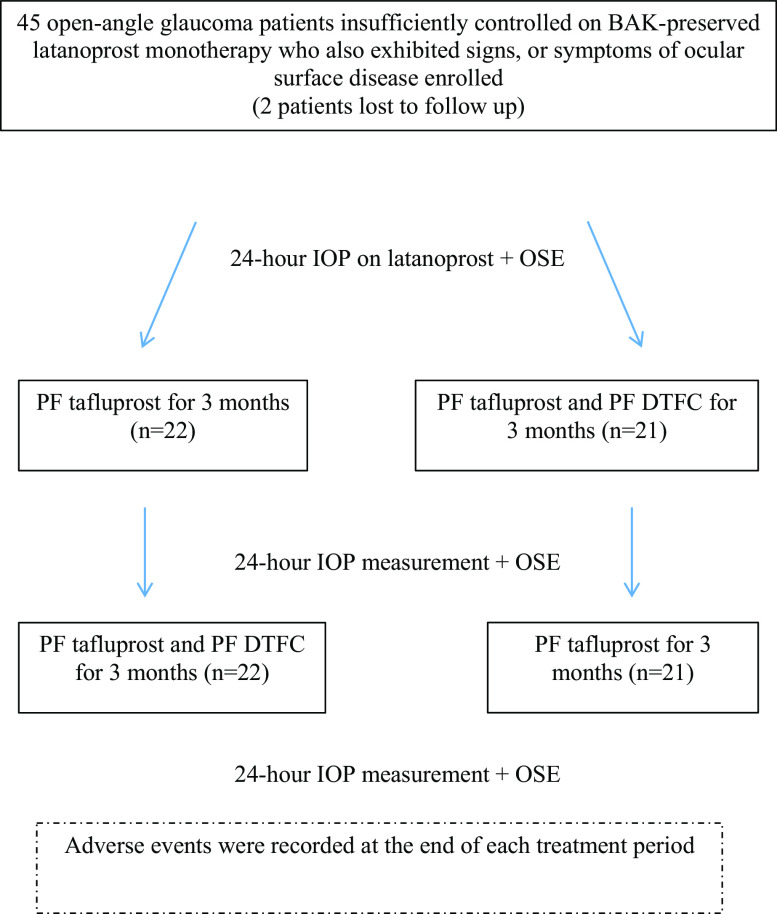
Flowchart of the study. *BAK* benzalkonium chloride, *DTFC* dorzolamide/timolol fixed combination, *OSE* ocular surface evaluation

Forty-three open-angle glaucoma patients (22 women and 21 men) completed the study out of 45 enrolled (Fig. 1). There were 24 patients with exfoliative glaucoma, 18 with primary open-angle glaucoma, and one with pigmentary glaucoma. The mean ± SD age of participants was 66.4 ± 12.3 years. Sixteen patients were using generic latanoprost whereas 27 were using branded latanoprost.

### Intraocular Pressure

**Fig. 2:**
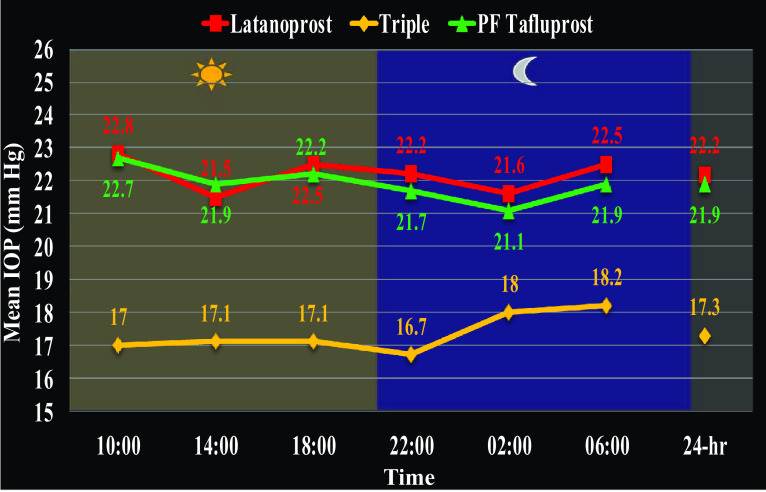
24-h IOP control with BAK-preserved latanoprost (*red*), preservative-free tafluprost (*green*), and combined PF triple therapy (tafluprost and dorzolamide/timolol fixed combination) (*yellow*)

The mean untreated morning IOP of the study cohort was 30.6 mmHg and the mean 24-h latanoprost-treated baseline IOP was 22.2 ± 2.9 mmHg (Table 1). PF tafluprost obtained lower IOP compared to latanoprost at two night-time points: 02:00 (*P* = 0.027) and 06:00 (*P* < 0.001) (Table 1, Fig. 2). Moreover, PF tafluprost reduced mean 24-h IOP to a greater extent than preserved latanoprost (21.9 vs 22.2 mmHg; *P* = 0.006). A greater reduction with PF tafluprost was also established with regard to peak 24-h IOP (23.9 vs 24.5 mmHg; *P* = 0.001) and 24-h IOP fluctuation (3.9 vs 4.6 mmHg; *P* = 0.001); (Table 1, Fig. 2).

**Table 1:** Efficacy comparisons between preserved latanoprost and PF tafluprost

Time/IOP parameter	Latanoprost (mmHg)	Tafluprost (mmHg)	*P* value
06:00	22.5 ± 3.8	21.9 ± 3.7	<0.001*
10:00	22.8 ± 3.0	22.7 ± 3.6	1.0*
14:00	21.5 ± 2.6	21.9 ± 3.0	0.468*
18:00	22.5 ± 3.2	22.2 ± 3.4	1.0*
22:00	22.2 ± 3.9	21.7 ± 3.8	0.126*
02:00	21.6 ± 3.2	21.1 ± 3.5	0.027*
Mean 24-h IOP	22.2 ± 2.9	21.9 ± 3.2	0.006
Trough 24-h IOP	19.9 ± 2.7	20.1 ± 2.8	0.421
Peak 24-h IOP	24.5 ± 3.3	23.9 ± 3.5	0.001
24-h fluctuation	4.6 ± 1.6	3.9 ± 1.3	0.001

Results are presented as mean ± standard deviation

* Bonferroni-adjusted *P* values

Triple PF therapy, comprising PF tafluprost and PF DTFC, provided significantly better 24-h IOP characteristics than preserved latanoprost or PF tafluprost monotherapies. Compared to preserved latanoprost the triple regimen significantly reduced IOP at all individual time points, mean 24-h IOP (17.3 vs 22.2 mmHg), trough 24-h IOP (15.3 vs 19.9 mmHg), and peak 24-h IOP (19.8 vs 24.5 mmHg) (all *P* < 0.001; Table 2, Fig. 2). A similar picture emerged when the triple PF therapy was compared with PF tafluprost (Table 3, Fig. 2).

**Table 2:** Efficacy comparisons between preserved latanoprost baseline and triple PF therapy regimen (tafluprost and dorzolamide/timolol fixed combination)

Time/IOP parameter	Latanoprost (mmHg)	Triple therapy (mmHg)	*P* value
06:00	22.5 ± 3.8	18.2 ± 3.7	<0.001*
10:00	22.8 ± 3.0	17.0 ± 3.1	<0.001*
14:00	21.5 ± 2.6	17.1 ± 2.8	<0.001*
18:00	22.5 ± 3.2	17.1 ± 3.2	<0.001*
22:00	22.2 ± 3.9	16.7 ± 3.1	<0.001*
02:00	21.6 ± 3.2	18.0 ± 3.0	<0.001*
Mean 24-h IOP	22.2 ± 2.9	17.3 ± 2.7	<0.001
Trough 24-h IOP	19.9 ± 2.7	15.3 ± 2.6	<0.001
Peak 24-h IOP	24.5 ± 3.3	19.8 ± 3.4	<0.001
24-h fluctuation	4.6 ± 1.6	4.4 ± 2.3	0.726

Results are presented as mean ± standard deviation

* Bonferroni-adjusted* P* values

**Table 3:** Efficacy comparisons between PF tafluprost and triple PF therapy (tafluprost and dorzolamide/timolol fixed combination)

IOP parameter	Tafluprost (mmHg)	Triple therapy (mmHg)	*P* value	IOP change (%)
06:00	21.9 ± 3.7	18.2 ± 3.7	<0.001*	−16.9
10:00	22.7 ± 3.6	17.0 ± 3.1	<0.001*	−25.2
14:00	21.9 ± 3.0	17.1 ± 2.8	<0.001*	−22.0
18:00	22.2 ± 3.4	17.1 ± 3.2	<0.001*	−23.0
22:00	21.7 ± 3.8	16.7 ± 3.1	<0.001*	−23.1
02:00	21.1 ± 3.5	18.0 ± 3.0	<0.001*	−14.7
Mean 24-h IOP	21.9 ± 3.2	17.3 ± 2.7	<0.001	−21.1
Trough 24-h IOP	20.1 ± 2.8	15.3 ± 2.6	<0.001	−23.9
Peak 24-h IOP	23.9 ± 3.5	19.8 ± 3.4	<0.001	−17.2
24-h fluctuation	3.9 ± 1.3	4.4 ± 2.3	0.04	+12.8

Results are presented as mean ± standard deviation

* Bonferroni-adjusted *P* values

**Fig. 3:**
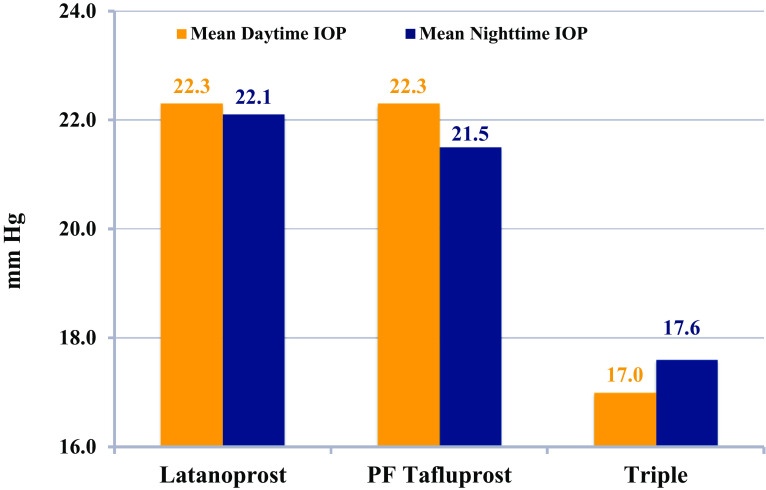
Daytime (*orange*) and nighttime (*blue*) efficacy of the study medications

It is worth noting that both prostaglandin monotherapies achieved identical mean daytime IOP (22.3 mmHg) (Fig. 3). However, the reason for the statistical difference between PF tafluprost and latanoprost in this study was due to a greater nighttime efficacy of PF tafluprost (21.5 vs 22.1 mmHg; *P* < 0.001). Finally, the triple PF therapy regimen offered significantly lower daytime and nighttime IOP than either preserved latanoprost or PF tafluprost (*P* < 0.001 for all comparisons).

### Ocular Surface Assessment

Overall, PF tafluprost was associated with significantly better ocular surface parameters than preserved latanoprost (TFBUT, 6.7 vs 6.0; corneal stain, 1.3 vs 2.2, Schirmer I test, 9.1 vs 8.2, respectively; *P* < 0.01 for all comparisons) (Table 4). Triple therapy was statistically similar to latanoprost with regard to TFBUT and Schirmer tests but was significantly better than latanoprost with regard to corneal staining (1.7 vs 2.2; *P* < 0.001) (Table 4). On the other hand, PF tafluprost monotherapy demonstrated significantly better ocular surface parameters compared to triple PF therapy (*P* < 0.01 for all comparisons) (Table 4). These findings suggest that PF tafluprost monotherapy is associated with a healthier ocular surface as opposed to either BAK-preserved latanoprost monotherapy or a triple PF regimen. The latter observation is not surprising since a triple PF regimen can have a more negative impact upon the ocular surface than a PF monotherapy as a result of the effect of multiple instillations with three different active ingredients compared with only one.

**Table 4:** Comparison of ocular surface signs with preserved latanoprost, PF tafluprost, and triple PF therapy (tafluprost and dorzolamide/timolol fixed combination)

Ocular surface parameter	Latanoprost (mean ± SD)	PF tafluprost (mean ± SD)	Triple PF therapy (mean ± SD)
Corneal stain (van Bijsterveld score)	2.2 ± 0.7	1.3 ± 0.6*	1.7 ± 0.6*
Schirmer test (mm)	8.2 ± 4.7	9.1 ± 4.4*	8.2 ± 4.5
Break-up time (s)	6.0 ± 2.2	6.7 ± 2.2*	6.1 ± 2.3

An asterisk denotes a statistically significant difference (*P* < 0.001) vs latanoprost baseline. All three ocular surface parameters were also significantly better (*P* < 0.001) with PF tafluprost vs triple PF therapy

*SD* standard deviation

### Adverse Events

All three study regimens were well tolerated (Table 5). It is worth noting, however, that PF tafluprost demonstrated a significantly smaller mean number of adverse events (0.77) than either preserved latanoprost (1.42) or the triple PF therapy regimen (1.37) (*P* < 0.001 for both comparisons). The mean number of adverse events observed with preserved latanoprost was statistically similar to that seen with triple PF therapy (*P* = 0.65). Specifically, study subjects reported a significantly greater prevalence of tired eyes (9.3%) or fluctuating vision (9.3%) with preserved latanoprost compared to PF tafluprost (0%) or triple PF therapy (0%) (*P* = 0.018 for both comparisons; Table 5). Conversely, burning (6.9%), stinging (20.9%), and bitter taste (11.6%) were significantly more common with the triple PF therapy regimen than either latanoprost or tafluprost monotherapies.

**Table 5:** Comparison of the most clinically important/commonest adverse events recorded with preserved latanoprost, PF tafluprost, and triple PF therapy (tafluprost and dorzolamide/timolol fixed combination)

Adverse event	Latanoprost *n* (%)	Tafluprost *n* (%)	Triple therapy *n* (%)	*P* value
Tired eyes	4 (9.3)	0 (0)	0 (0)	0.018
Fluctuating vision	4 (9.3)	0 (0)	0 (0)	0.018
Burning	0 (0)	0 (0)	3 (6.9)	0.05
Stinging	2 (4.6)	3 (6.9)	9 (20.9)	0.028
Bitter taste	0 (0)	0 (0)	5 (11.6)	0.007
Hyperemia	8 (18.6)	5 (11.6)	4 (9.3)	0.074
Itchiness	5 (11.6)	3 (6.9)	1 (2.3)	0.091
Ocular ache	2 (4.6)	0 (0)	0 (0)	0.135
Blurring of vision	4 (9.3)	3 (6.9)	2 (4.6)	0.368
Total number of adverse events	56	34	55	<0.001

## Discussion

To the best of our knowledge this is the first study to compare the 24-h efficacy and ocular surface status with a triple PF regimen versus a popular monotherapy: BAK-preserved latanoprost in a cohort of open-angle glaucoma patients. This study also evaluated the same parameters after switching from preserved latanoprost to PF tafluprost monotherapy. It should be noted that all participants were insufficiently controlled on preserved latanoprost monotherapy and also exhibited signs or symptoms of OSD. We opted for a complete 24-h IOP assessment, as previous research has shown conclusively that such studies offer a comprehensive evaluation of the true efficacy of available treatment options [29–32].

Lifelong topical antiglaucoma therapy with preserved medications has a detrimental effect on ocular surface health, especially when multiple agents are used. Currently, there is limited controlled evidence pertaining to the health of the ocular surface with chronic combined antiglaucoma therapy [33]. In the present study we employed three easy-to-perform clinical ocular surface metrics: the TFBUT, corneal staining, and the Schirmer test. Although these tests may at times show suboptimal consistency and reliability they are still the mainstay of clinical tests used to detect and quantify epithelial and tear film abnormalities [34].

The 24-h efficacy results of the present trial first suggest that switching from preserved latanoprost to PF tafluprost monotherapy in glaucoma patients with symptoms/signs of OSD attains a statistically significant IOP reduction at the 06:00 and 02:00 time points as well as for the mean, peak, and fluctuation of 24-h IOP. The comparison between mean daytime and nighttime IOP control with the two prostaglandins highlights the point that while both medications obtain identical daytime IOP (22.3 mmHg) PF tafluprost is more efficient at night (21.5 vs 22.1 mmHg; *P* < 0.001). This observation is consistent with previous 24-h evidence indicating that PF tafluprost displays superior nighttime efficacy compared to latanoprost [35]. In our study, the 24-h efficacy difference between preserved latanoprost and PF tafluprost was 0.3 mmHg. Although this efficacy difference was statistically significant it may not be clinically meaningful. This is because the potential clinical value of a 24-h efficacy difference remains to be elucidated. On the other hand, there may be a long-term advantage because PF tafluprost provided significantly lower peak 24-h IOP and a significantly narrower 24-h IOP fluctuation. There is emerging evidence that these 24-h parameters (especially peak 24-h IOP) are associated with a better long-term prognosis [36–41].

Triple PF regimen demonstrated superior efficacy compared to the two prostaglandins at all individual time points, and for the mean, peak, and trough 24-h IOP (all *P* < 0.001). Since the observed 24-h difference is considerable (compared to latanoprost baseline a 4.9 mmHg or 22.1% reduction) it is clear that such an efficacy difference is not only statistically significant but also clinically meaningful in the stepwise management of glaucoma. Thus, the results of the present study suggest that when monotherapies are insufficient and a substantial IOP lowering is needed the triple PF regimen employed herein may represent a suitable option to attain good efficacy together with good tolerability.

As expected, the triple PF regimen was also significantly more efficacious than PF tafluprost monotherapy for all comparisons except 24-h fluctuation. It is of interest that PF tafluprost monotherapy achieved a significantly lesser 24-h IOP fluctuation than the triple PF regimen (3.9 vs 4.4 mmHg;* P* = 0.04). This could be due to a less uniform pattern of IOP reduction by DTFC. Owing to the presence of timolol, the additional ocular hypotensive effect was more pronounced during the day than during the night, thus generating a greater fluctuation of treated 24-h IOP. This confirms previously published 24-h evidence [29, 42] that shows that 24-h IOP fluctuation with combined therapy is either the same or slightly worse than that obtained with prostaglandin monotherapies. Moreover, the present study highlights the fact that the true efficacy of PF tafluprost and the triple PF regimen would have remained undetected without a complete 24-h evaluation. Since 24-h monitoring is impractical for the vast majority of glaucoma patients in routine care, we should rely on published controlled 24-h evidence to facilitate management decisions in glaucoma therapy [29–32, 35].

A key consideration beyond efficacy in chronic, asymptomatic diseases like glaucoma is the long-term tolerability of glaucoma therapies. Long-term tolerability can affect adherence, efficacy, and ultimately therapeutic success and prognosis. Consequently, the long-term impact of glaucoma medications on ocular surface health should be taken into account in all therapeutic algorithms [6, 10, 13]. In the present trial a similar total number of adverse events was observed with preserved latanoprost and triple PF therapy. In contrast, significantly fewer adverse events were recorded with PF tafluprost. These results suggest that PF treatment options can certainly improve tolerability in a group of glaucoma patients with symptoms or signs of OSD. To monitor the health of the ocular surface with the three regimens in our study, we employed three popular metrics (TFBUT, corneal stain, and Schirmer test). Compared to BAK-preserved latanoprost, PF tafluprost was associated with significantly better scores in all three tested parameters, indicating a significant improvement in terms of ocular surface health.

On the other hand, the triple PF regimen demonstrated significantly less corneal staining score and similar TFBUT and Schirmer test scores compared to BAK-preserved latanoprost. It was surprising that a combination of three PF daily drops containing three different active ingredients demonstrated a similar ocular surface profile and comparable tolerability to a well-known reference prostaglandin monotherapy (i.e., preserved branded or generic latanoprost). This is likely due to the elimination of BAK, but could also be attributed to the relatively small sample size (43 patients) or the short duration of the study (3 months). Moreover, to better reflect clinical practice the study cohort was preselected for having symptoms or signs of OSD and showing insufficient IOP control with latanoprost monotherapy. Nevertheless, it is well established that the majority of glaucoma patients demonstrate OSD and most glaucoma patients will require adjunctive therapy [1, 6, 9, 13].

The triple PF regimen employed in this study can conceivably be considered a logical maximal medical therapy option for many patients. This implies that to succeed with long-term therapy such an option should be as tolerable as possible. It should be emphasized, however, that the short-term tolerability of antiglaucoma medications does not necessarily mirror long-term tolerability. In fact, it is reasonable to assume that in contrast to PF treatment options, the tolerability of BAK-containing medications would decrease over the long term because of the cumulative toxic effect of the preservative on the ocular surface.

Certain limitations of this study need to be taken into account. Firstly, we did not employ sophisticated and possibly more accurate metrics for the evaluation of the ocular surface. Instead, to better reflect standard clinical practice worldwide, we opted for the three most commonly used tests in daily practice (i.e., TFBUT, corneal stain, and Schirmer I test). Promising research avenues include assessment of patient symptoms with validated questionnaires, tear film quality and quantity valuation by means of osmolarity measurements, and assessment of the ocular surface damage with lissamine green. Ideally, meibomian gland morphology and function tests should also be included [26, 27]. Finally, once validated, novel tests such as the measurement of matrix metalloproteinase-9 (MMP-9) [43], ocular surface epithelium impression cytology [44], and electronic assessment of tear film properties [45], which objectively characterize the tear film and image in vivo ocular surface tissues, may prove instrumental in future research [46]. Another limitation of the present study is that we did not evaluate the long-term benefits or the health outcome with PF medications. The detection of significant differences, however, in terms of 24-h efficacy and ocular surface health with the short-term use of the PF medications investigated herein, lends support to the hypothesis that clear-cut differences should emerge over the long term, too. This assumption requires further validation. Lastly, this study included glaucoma patients insufficiently controlled with preserved branded or generic latanoprost who exhibited signs or symptoms of OSD. It remains unclear what impact (if any) the inclusion of glaucoma patients treated with generic latanoprost formulations (four formulations employed in 16/43 of our patients) had. This approach, however, was selected to reflect current clinical practice.

Overall the present investigation established that in glaucoma patients with symptoms or signs of OSD insufficiently controlled on preserved latanoprost, PF tafluprost provides greater 24-h efficacy and enhances ocular surface health and tolerability. A triple PF regimen comprising tafluprost and DTFC provides superior 24-h IOP control compared with preserved latanoprost or PF tafluprost. The ocular surface profile of the triple PF regimen was found to be similar to that of BAK-preserved latanoprost.

## Conclusion

In the present crossover study eligible glaucoma patients who demonstrated symptoms or signs of OSD on preserved branded or generic latanoprost were switched to PF tafluprost therapy and a triple PF regimen consisting of PF tafluprost and PF DTFC. Treatment with PF tafluprost not only offered statistically lower mean, peak, and fluctuation of 24-h IOP but also enhanced ocular surface parameters and tolerability. The triple PF regimen provided significantly better 24-h IOP control (−22%) than latanoprost baseline therapy. These results suggest that PF treatment options can meaningfully improve tolerability in glaucoma patients with symptoms or signs of OSD.
